# Left Ventricular-Arterial Coupling as an Independent Predictor of Adverse Events in Young Patients with ST Elevation Myocardial Infarction—A 3D Echocardiographic Study

**DOI:** 10.3390/biomedicines12010105

**Published:** 2024-01-04

**Authors:** Alina Ioana Scarlatescu, Miruna Mihaela Micheu, Ioana Gabriela Petre, Nicoleta Oprescu, Ana Maria Mihail, Ioana Denise Cojocaru, Radu Gabriel Vatasescu

**Affiliations:** 1 Department of Cardiology, Clinic Emergency Hospital of Bucharest, Calea Floreasca 8, 014461 Bucharest, Romania; alina.scarlatescu@gmail.com (A.I.S.); i_comanescu@yahoo.com (I.G.P.); nicoleta_m_oprescu@yahoo.com (N.O.); mihailanamaria94@yahoo.com (A.M.M.); ioana.cojocaru@otlook.com (I.D.C.); radu_vatasescu@yahoo.com (R.G.V.); 2 Department IV—Cardio-Thoracic Pathology, Carol Davila University of Medicine and Pharmacy, Eroii Sanitari Bvd. 8, 050474 Bucharest, Romania

**Keywords:** STEMI, MACE, ventricular-arterial coupling, 3D echocardiography

## Abstract

Left ventricular-arterial coupling (VAC) is a key determinant of global cardiovascular performance, calculated as the ratio between arterial elastance (EA) and left ventricular end-systolic elastance (EES). Over the years, acute myocardial infarction (STEMI) has remained an important cause of morbidity and mortality worldwide. Although, until recently, it was considered a disease occurring mostly in older patients, its prevalence in the young population is continuously rising. In this study, we aimed to investigate the role of 3D VAC and its derived indices in predicting adverse outcomes in young patients with STEMI. We prospectively enrolled 84 young patients (18–51 years) with STEMI who underwent primary PCI and 28 healthy age and sex-matched controls. A 3D echocardiography was used for non-invasive measurements of end-systolic elastance (EES), arterial elastance (EA), and VAC (EA/EES). The occurrence of major adverse cardiac events (MACE) was assessed one year after the index STEMI. Out of 84 patients, 15.4% had adverse events at 12 months follow-up. Patients were divided into two groups according to the presence or absence of MACE. There were no significant differences in arterial elastance between the two groups. EA was higher in the MACE group but without statistical significance (2.65 vs. 2.33; *p* = 0.09). EES was significantly lower in the MACE group (1.25 ± 0.34 vs. 1.91 ± 0.56. *p* < 0.0001) and VAC was higher (2.2 ± 0.62 vs. 1.24 ± 0.29, *p* < 0.0001). ROC analysis showed that VAC has a better predictive value for MACE (AUC 0.927) compared with EA or EEA but also compared with a classical determinant of LV function (LVEF and LVGLS). A VAC value over 1.71 predicts unfavourable outcome with 83.3% sensitivity and 97.1% specificity. In both univariate and multivariate COX regression analysis, VAC remained an independent predictor for MACE and demonstrated incremental prognostic value over LVEF and LVGLS in the proposed statistical models. In conclusion, 3D VAC is an independent predictor of adverse events in young patients with STEMI at a 12 month follow-ups and could be used for a more accurate risk stratification in the acute phase.

## 1. Introduction

Acute ST-elevation myocardial infarction (STEMI) remains a leading cause of morbidity and mortality worldwide despite continuous advances in diagnostic, prognostic, and therapeutic methods [1].

Early identification of patients with a potential unfavourable outcome after acute myocardial infarction (AMI) might lead to a more accurate risk stratification [2]. The use of prognostic markers could better identify patient groups at risk that would benefit from more intensive treatment or preventive strategies [3,4]. Echocardiography plays an important role in risk stratification and prognosis assessment after myocardial infarction [5]. There have been many attempts to predict the risk of unfavourable outcomes in patients with myocardial infarction, and to this day many echocardiographic parameters (e.g., left ventricular ejection fraction—LVEF, ventricular volumes, wall motion score index—WMSI, mitral regurgitation, and LA volume) and biomarkers have been tested in the search for the perfect prognostic marker or combination thereof [5,6,7,8]. Although the results of recent studies are promising, no ideal prognostic marker has yet been identified.

VAC represents the interaction between the ventricle and the arterial system, reflecting global cardiac performance [9]. Left ventricular-arterial coupling is calculated as the ratio between arterial elastance (EA), as an expression of afterload, and ventricular end-systole elastance (EES), being a measure of left ventricle (LV) myocardial performance independent of ventricular filling pressure [10]. The gold standard for measuring VAC is the invasive measurement of ventricular volumes and pressures to assess pressure-volume loops and to calculate EA and EES, which is not feasible in daily clinical practice [11]. EES is a characteristic of cardiac function, contractility, and morphology, being independent of preload and afterload, while the arterial load represents all extracardiac forces as opposed to left ventricular ejection, being the arterial afterload [12]. An alteration of VAC is a marker of disease severity and is associated with outcome [13]. According to the latest ESC consensus on the role of VAC in cardiovascular disease [9], normal values for EA and EES are 2.2 ± 0.8 mmHg/mL and 2.3 ± 1.0 mmHg/mL, respectively, and for VAC 1 ± 0.36 mmHg/mL. Therefore, when EA/EES is approximately equal to 1 (0.7–1), the LV and arterial system are optimally coupled to produce efficient mechanical work [14]. If EA/EES < 1.0, the mechanical work per stroke volume remains close to the optimal values; as it increases above 1, the mechanical work decreases significantly, and the ventricle becomes less efficient [10]. In practice, EES indicates how much end-systolic volume increases and how much stroke volume decreases in response to increased end-systolic pressures.

In recent years, 3D echocardiography has emerged as a more accurate measurement of left ventricular volumes and ejection fraction; other advantages compared to 2D echocardiography lie in its better precision and reproducibility for volume measurement and therefore for VAC assessment [15,16]. In this study, we aimed to evaluate the prognostic value of VAC determined by 3D echocardiography in young patients with STEMI.

## 2. Materials and Methods

### 2.1. Study Population

This prospective study was conducted in the Department of Cardiology of the Clinical Emergency Hospital of Bucharest between 2019 and 2021. The study group consisted of 84 young patients (aged between 18 and 51 years old) with a first STEMI, admitted to our hospital between 2019 and 2020, and treated by primary PCI. We also selected a control group of 28 age and sex-matched healthy volunteers for validation of the results.

All included patients received optimal medical therapy according to current clinical practice guidelines [17]. We excluded patients with a previous myocardial infarction or cardiac surgery, structural cardiac disease of severe pre-existing valvular heart disease, recent surgery or trauma (within 6 months), severe respiratory/hepatic/renal failure, active malignancy or autoimmune diseases, active infections, pregnancy, patients with addictions, poor compliance, or those who refused to sign the informed consent. The study was approved by the Medical Ethics Committee of the Bucharest Emergency Clinical Hospital. All patients signed informed consent at enrolment.

### 2.2. Study Protocol

Baseline evaluation at enrolment (T0) included gathering demographic data, physical examination, clinical parameters, laboratory data, angiographic data, and echocardiographic features.

Standard transthoracic echocardiography was performed in all patients using the GE VIVID E95 ultrasound system. Recorded data was analysed offline using Echo PAC workstations. Image acquisition and measurements were performed by two different experienced sonographers according to the international guidelines of the European Association of Cardiovascular Imaging [18] and the American Society of Echocardiography [19]. In addition to the conventional parameters, we also determined the LV global longitudinal strain (LV GLS), 3D left ventricular volumes, and 3D LVEF. 

**Figure 1:**
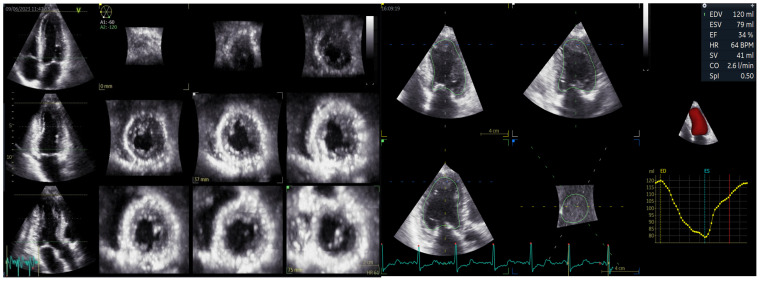
Measurements of left ventricular volumes and ejection fraction with three-dimensional echocardiography using the 4D auto LVQ software Vivid GE E95 System.

Three-dimensional echocardiography data set acquisition of the LV was performed by the same examiner at the end of the standard 2D examination using a dedicated transducer. A full-volume scan was acquired from an apical approach during an end-expiratory apnoea [13]. Quantification of 3D LV parameters was performed using commercially available semi-automated software (GE Vivid 4D auto LVQ, EchoPAC version 204). The software automatically delineates the LV endocardial border in a 3D model from the end-diastolic and end-systolic phases, offering a 3D model of the left ventricle. LV end-diastolic volume (LVEDV), end-systolic volume (LVESV) and EF, stroke volume (SV), and cardiac output (CO) are finally displayed (Figure 1). 

Based on 3D volumes and 3D EF, we determined 3D left ventricular-arterial coupling (3D VAC). VAC was calculated as the ratio between arterial elastance and end-systolic left ventricular elastance. The following detailed formulas were used (adapted for 3D echocardiography after Chen et al. [9,20]):

Arterial elastance (EA) was defined as the ratio of end-systolic pressure and stroke volume (ESP/SV). End-systolic pressure (ESP) was determined as 0.9 × systolic arterial blood pressure [9,20].

EA = ESP/SV = (0.9 × systolic BP)/3D SV.

EES = [diastolic BP − (ENd(est) × systolic BP × 0.9)]/(ENd(est) × 3D SV)

ENd (est) = 0.0275 − 0.165 × 3D LVEF + 0.3656 × (diastolic BP/(systolic BP × 0.9)) + 0.515 × End (avg).

End (avg) = 0.35695 − 7.2266 × tNd + 74.249 × t Nd^2^ − 307.39 × tNd^3^ + 684.54 × tNd^4^ − 856.92 × tNd^5^ + 571.95 × tNd^6^
− 159.1 × tNd^7^

**Figure 2:**
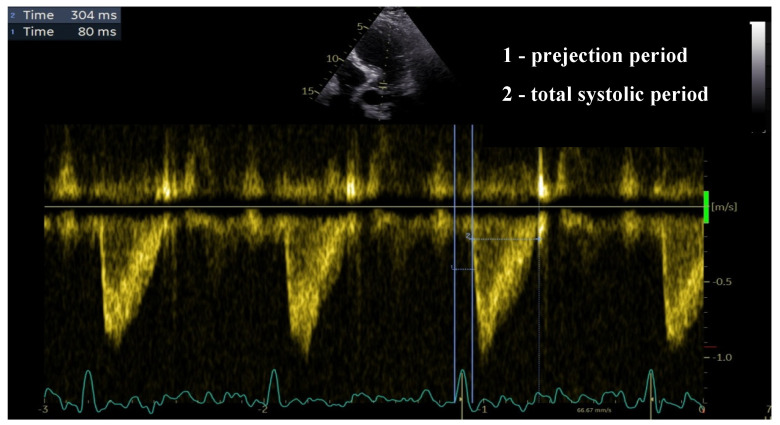
Calculation of tND as the ratio between the pre-ejection period and total systolic period.

ENd (est) is the estimated normalized ventricular elastance at the onset of ejection and tNd is the ratio of the pre-ejection period (R wave to flow onset) to total systolic period (R wave to end of systolic flow)—measured using aortic PW doppler, as shown in Figure 2.

All included patients also underwent coronary angiography at hospital admission according to current clinical practice guidelines [17].

### 2.3. Follow-Up—Primary Endpoint

Patients were followed up for at least one year after STEMI. At 12 months (T12), a telephone follow-up was performed due to COVID-19 pandemic restrictions. The primary endpoint was the occurrence of MACE, defined as death from cardiovascular causes, heart failure requiring hospital admission, or repeat PCI/CABG due to ischemia/infarction (in concordance with previous trials) [21].

### 2.4. Statistical Analysis

Statistical analysis was performed using SPSS (IBM SPSS Statistics v.22.0, IBM Corp., Armonk, NY, USA) and GraphPad software (GraphPad Prism 9.0.0, San Diego, CA, USA). Kolmogorov–Smirnov and Mann–Whitney U-tests were used to assess the distribution of the data. Categorical variables were presented as percentages, while continuous data were presented as means ± standard deviation. In order to compare the variables between them, we used Student T and Chi-square tests. ROC analysis (receiver operating curve) was used to determine the AUC (area under the curve), which represents the predictive power of the parameters; we also determined cut-off values for the significant prognostic parameters using the Youden index. We also performed COX univariate and multivariate regression analysis to identify significant independent predictors for MACE. Kaplan-Meier analysis was then used to represent the differences between groups and the occurrence of MACE. *p* < 0.05 was considered statistically significant.

## 3. Results

### 3.1. Patient Characteristics

Eighty-four young patients with STEMI were included and followed up for 12 months. Baseline demographic and clinical characteristics for the entire cohort, according to the presence/absence of MACE, are summarized in Table 1. 

At discharge, all patients received optimal medical therapy (administration of dual antiplatelet therapy, beta-blockers, angiotensin convertor enzyme inhibitors/angiotensin receptor blockers, and statins) in agreement with current clinical practice guidelines [17]. Regarding cardiovascular risk factors in the study group, 86% were males, 45.2% were hypertensives, 14.1% had diabetes, 86.9% were smokers, and 86.9% had dyslipidaemia.

Patients were divided into two groups: MACE and non-MACE in accordance with the presence or absence of adverse events at one year follow-up. Medical treatment did not differ significantly between the two groups (Table 1).

Thirteen out of 84 patients (15.4%) recorded an adverse event during the follow-up period of 12 months, as follows: 2 (15%) died—death of cardiovascular causes, 7 (53%) required hospital admission for decompensated heart failure, and 4 (30%) suffered a new acute coronary syndrome also requiring hospital admission and coronary angiography reassessment. There were no significant differences between groups regarding the angiographic characteristics: the culprit vessel, the presence of multivessel CAD, or time from onset to balloon. Laboratory characteristics were also almost the same between groups except for CK-MB levels that were significantly higher in the MACE group (*p* = 0.02).

### 3.2. Echocardiographic Parameters

Echocardiography was performed at baseline (T0). We have detailed below (Table 2) the echocardiographic parameters recorded in the study group of young patients with STEMI, divided according to the presence or absence of the MACE, and in the control group (healthy volunteers). 

The mean LVEF in the study group was 42.19 ± 7.34. Patients with MACE at follow-up had significantly higher end-systolic and end-diastolic ventricular volumes (indexed 2D LVEDV 63.96 ± 14.48 mL/mp vs. 51.16 ± 12.18 mL/mp, *p* = 0.002; Indexed 2D LVESV 43.98 ± 14.48 mL/mp vs. 28.47 ± 8.47 mL/mp, *p* = 0.001; Indexed 3D LVEDV 68.29 ± 13.14 mL/mp vs. 55.22 ± 13.14 mL/mp, *p* = 0.002; 3D indexed LVESV 46.28 ± 12.49 mL/mp vs. 30.65 ± 9.03 mL/mp, *p* = 0.001), more impaired LVGLS (−8.9 ± 2.51 vs. −13.95 ± 3.066, *p* < 0.0001) and lower ejection fraction (2D LVEF: 35.75 ± 5.81% vs. 43.61 ± 6.608%, *p* < 0.0001; 3D LVEF: 37.16 ± 6.307% vs. 44.88 ± 6.605%, *p* < 0.0001) at baseline. Mechanical dispersion was also greater in the MACE group (88.28 ± 27.67 ms vs. 65.25 ± 24.13 ms, *p* = 0.004). Right ventricular function was similar between the two groups.

Regarding diastolic function, no significant differences were observed between E and A waves or the E/A ratio, but filling pressures expressed by the mean E/e’ ratio were significantly higher in the MACE group (10.68 ± 7.59 vs. 7.59 ± 2.03, *p* < 0.0001) as well as 2D LA maximum volume (29.82 ± 10.77 mL/mp in MACE group vs. 22.85 ± 6.08 mL/mp in the non-MACE group, *p* = 0.027).

### 3.3. EA, EES and VAC

In the control group, we obtained lower values for VAC (0.8 ± 0.23 control vs. 1.37 ± 0.48 STEMI) and higher values for EES (2.3 ± 0.7 control vs. 1.85 ± 0.57 STEMI), but almost similar values for EA (2.1 ± 0.6 control vs. 2.38 ± 0.59 STEMI) compared to the study group. VAC values lower than 1 in the control group suggest an optimal coupling between LV and arterial system as expected since these are healthy subjects.

**Figure 3:**
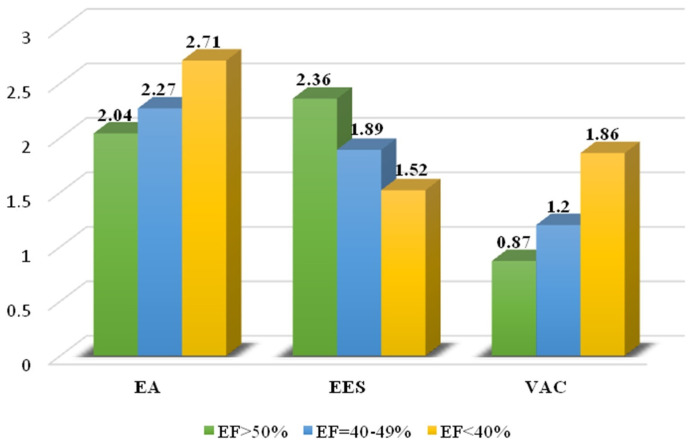
EA, EES, and VAC according to the degree of LV systolic dysfunction.

We divided the study population into 3 groups, according to the degree of LV systolic dysfunction, as follows: preserved EF (>50%), mildly reduced EF (FE 40–49%), and reduced EF (<40%)—in accordance with the classification of heart failure from the latest ESC guidelines [22]. We can observe that with the decrease in LV systolic function, LVGLS and EEA decrease progressively, while VAC and EA increase. The lowest VAC values were encountered in the group with preserved EF, a higher value was in the second group, and the highest values of VAC were observed in the group with reduced EF (Figure 3). Comparing left ventricular-arterial coupling parameters with LVEF, we observed that patients with severe systolic dysfunction had the lower EEA values and the highest VAC.

Regarding the correlations between VAC and other echocardiographic variables, we observed strong correlations with LV function parameters. VAC positively correlated with LVGLS (r = 0.785, *p* < 0.0001) and mechanical dispersion (r = 0.287, *p* = 0.008) and inversely correlated with 2DLVEF (r = −0.917, *p* < 0.0001) and 3DLVEF (r = −0.943, *p* < 0.0001). Correlations for EA, EES, and VAC are depicted in Table S1.

### 3.4. VAC and MACE

There were no significant differences in arterial elastance between the two groups (MACE vs. non-MACE). EA was higher in the MACE group but without statistical significance (2.65 vs. 2.33. *p* = 0.09). EES was significantly lower in the MACE group (1.25 ± 0.34 vs. 1.91 ± 0.56. *p* < 0.0001) and VAC was higher (2.2 ± 0.62 vs. 1.24 ± 0.29. *p* < 0.0001)—Table S2.

Univariate COX logistic regression was used to identify the independent predictors for MACE in our study group. The following parameters proved to be associated with the occurrence of adverse events at one year follow-up: LVEF, LVEDV, LVESV, LVGLS, E/e’, and LA indexed maximum volume—Table 3.

**Figure 4:**
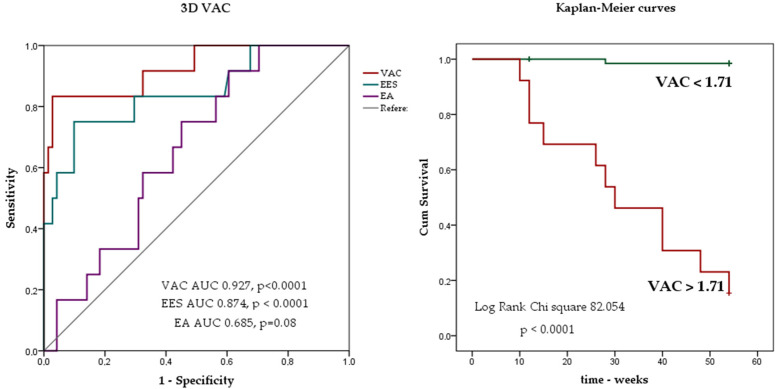
ROC analysis for VAC (**left**)—ROC curves for VAC (red), EES (blue), and EA (purple) Kaplan Meier analysis for VAC (**right**) showing the risk of MACE stratified by VAC cut-off value obtained from ROC analysis.

Among the newly tested parameters, EES and VAC, but not EA were found to be independent predictors of MACE in COX regression analysis. ROC analysis revealed that EA and EES alone had lower predictive value for MACE compared to their ratio (VAC): AUC for EES 0.847 (*p* < 0.0001). The AUC for EA was 0.658 (*p* = 0.08) and the AUC for VAC was 0.927 (*p* < 0.0001) (Figure 4 left). 

We compared the predictive value of the new variables with that of the parameters commonly used to assess systolic dysfunction and that are known predictors for adverse events after STEMI. VAC had a higher predictive value for MACE (AUC 0.927) compared with LVEF (AUC 0.861) or LVGLS (AUC 0.895).

A VAC value over 1.71 predicts unfavourable outcome with 83.3% sensitivity and 97.1% specificity. Kaplan Meier analysis confirmed that patients with EA/EES over the cut-off value had a worse outcome with Chi-square 92.67, *p* < 0.0001 as depicted in the figure below (Figure 4 right). Patients with VAC over 1.71 had lower LVEF, lower LVGLS values, higher ventricular volumes, E/e’, and LA volume values in the acute phase of STEMI.

In multivariate COX regression analysis, VAC > 1.71 proved to be an independent predictor for adverse events after STEMI in prediction models that included LAVi, VAC, LVEF, and E/e’. VAC maintained its prognostic value in all proposed multivariable COX regression models—Table 4. 

**Figure 5:**
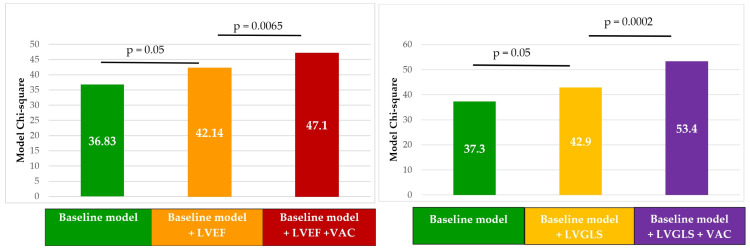
Graph showing the incremental prognostic value of VAC over traditional prognostic factors (the values represent the likelihood ratio χ^2^ test). Adding VAC over multivariable models containing LVEF (**left**) and LVGLS (**right**) led to a significant incremental prognostic power.

Moreover, VAC had incremental prognostic value over LVEF and LVGLS. Both regression models proved to have good discriminating power with AUC = 0.972, 95% CI (0.939–1), *p* = 0.000 for the first model (Figure 5 left) and AUC = 0.992, 95% CI (0.954–1), *p* = 0.000 for the second one (Figure 5 right).

### 3.5. Ventricular-Arterial Coupling by 2D and 3D Echocardiography 

For validation purposes of the adapted Chen’s formula, considering the fact that the original one used 2D echocardiography, we compared 2D with 3D VAC parameters. EA and VAC proved to be almost similar in 2D and 3D echocardiography; EES was lower in 3D echocardiography—Table 5.

## 4. Discussion

This study demonstrated that noninvasively measured 3D left ventricular arterial coupling is an independent predictor of unfavourable outcomes at 12 months follow-up in a group of young patients with a first STEMI. 

The clinical interest in ventriculo-arterial coupling is derived from its strong connection with cardiac energetics and efficiency [13]. The ability to deliver an efficient cardiac output is constantly challenged by beat-to-beat changes in hemodynamic conditions, contractility, and heart rate [9]. Acute changes in the hemodynamic and contraction frequencies of the left ventricle lead to the alteration of the global longitudinal and circumferential strain, and therefore, ventricular function [9]. Current methods of measuring LV function (LVEF, LVGLS) provide a partial assessment of the effects on the LV [9]. Non-invasive measurement of VAC may provide a comprehensive assessment of left ventricular performance and bring additional information beyond ejection fraction in the characterisation and clinical management of patients with CAD [9].

VAC is a key determinant of cardiovascular performance, calculated as the ratio between arterial elastance and ventricular elastance [23,24]. In this study, we determined the non-invasive VAC based on a formula proposed by Chen, which proved to be close to invasive measurements [11]. The novelty of our study is that we adapted the original formula using 3D LV measurements instead of 2D (6 beats vs. single beat), thus providing a more accurate estimation of EES. Experimental models showed that mechanical work is the highest when the EA/EES ratio is close to 1, while ventricular efficiency is maximal when the ratio is closer to 0.5 [23,25]. Ventricular-arterial uncoupling could therefore be a predictor for morbidity and mortality [9,26,27]. VAC is suboptimal in the presence of ventricular systolic dysfunction and EES is low secondary to pump dysfunction [28], an aspect also found in the present study. In our research, the mean EA/EES is increased, demonstrating impaired coupling, most likely due to an increase in both LV and arterial stiffness. We compared the VAC values of STEMI patients with a control group of healthy individuals, and we observed significantly lower values of VAC and EES in the study group. VAC values lower than 1 found in the control group are the expression of an optimal coupling between the LV and the arterial system, as expected in healthy individuals. 

By dividing our study group into three groups according to the degree of systolic dysfunction, we concluded that with the decrease in LV function that leads to a decrease in EES, a compensatory increase in EA occurs with the aim of maintaining an adequate intravascular volume and an optimal VAC.

VAC and its variations were studied in different physiological and pathological conditions. For example, in the elderly, there is an increase in both arterial and ventricular stiffness to maintain coupling within normal limits; however, cardiac reserve is reduced during exercise when this compensation is not possible anymore [29]. In the case of left ventricular dysfunction or heart failure, VAC is suboptimal, with EES being decreased secondary to pump dysfunction, while EA is increased due to increased impedance and decreased compliance [28]. There is even evidence that ventricular arterial uncoupling occurs before cardiac pump dysfunction [28]. Although myocardial ischemia and STEMI are the most common causes of ventricular dysfunction, this phenomenon can also be observed in other pathologies (e.g., DCM [30], HCM). A recent study [31] revealed that an acute increase in ventricular afterload causes the uncoupling between systole and diastole of the longitudinal strain curve. Secondly, there is a decrease in global longitudinal strain. An even greater uncoupling between systole and diastole was observed in older people. Furthermore, an increase in afterload secondary to increased aortic stiffness may further affect ventricular performance [32]. Increased pulse-wave velocity (PWV) has been linked to reduced coronary flow reserve in patients with CAD (even after successful revascularisation) and increased post-infarction NTproBNP [33,34]. Higher PWV values predict the occurrence of MACE in patients undergoing PCI. Increased stiffness of the aorta further affects ventricular function through inadequate coupling. Therefore, a non-invasive measurement of VAC may provide a comprehensive assessment of ventricular performance and may have incremental value over ejection fraction in the characterisation and clinical management of patients with CAD [9].

Regarding MACE prediction, in our study, VAC was significantly higher (2.2 ± 0.62 vs. 1.24 ± 0.29, *p* < 0.0001) and EES was significantly lower (1.25 ± 0.34 vs. 1.91 ± 0.56, *p* < 0.0001) in MACE group compared to the non-MACE group. EA was also higher in the MACE group but without statistical significance. ROC analysis confirmed the role of VAC in MACE prediction with an AUC of 0.927, higher than that of LVEF (AUC 0.861) and LVGLS (AUC 0.895). We also identified a cut of value of 1.7 for VAC (83.3% sensitivity and 97.1% specificity). Patients with VAC values over the cut-off had a higher rate of adverse events at follow-up compared to those with lower values. Similar results were obtained in a study on 41 patients with ischemic cardiomyopathy and moderate systolic dysfunction; in this trial patients with VAC values under 1.47 had a higher survival rate compared to those with impaired coupling (≥1.47) [25].

The severity of coronary involvement was associated with greater arterial stiffness [9]. The presence of CAD can affect LV systolic function, especially in its longitudinal axis. In another study of 891 patients with suspected CAD but with negative stress ultrasound and moderate systolic dysfunction, Bombardini et al. demonstrated that those with lower VAC reserve, measured as the difference between EA/EES at peak stress and rest, had a higher mortality rate compared to those with better VAC reserve [35].

In patients with heart failure with reduced ejection fraction, systemic tissue hypoperfusion occurs as systolic function and ventricular elastance decrease. In response, there is activation of the RAAS and the sympathetic nervous system, in an attempt to increase intravascular volume and arterial load to compensate for the altered systemic perfusion [26]; therefore, it increases EA. The increase in EA together with the adverse effects of excessive activation of the sympathetic system and the secondary increase in myocardial oxygen consumption creates a vicious circle that predisposes to further worsening of cardiovascular function [26]. Considering the above, VAC could also be useful for evaluating the clinical response to different therapeutic interventions in heart failure [36]. Suboptimal coupling due to an increase in ventricular-arterial stiffness also influences myocardial perfusion by increasing coronary flow in systole (up to 50%), exacerbating the impact of regional coronary ischemia in the event of impaired systolic performance [37]. ACEI/ARB treatment has been shown to improve both arterial and ventricular stiffness. In a cohort of 466 HF patients with low EF, a strong association was observed between VAC, NYHA class, natriuretic peptides, and adverse clinical events [27].

Considering the above we can say that 3D VAC holds great potential as a prognostic marker in our group of young patients with STEMI. Its use in clinical practice could contribute to the early identification of patients with potentially unfavourable outcomes and could lead to more accurate risk stratification, enabling a better outcome in the long term.

## 5. Limitations

This study included a relatively small number of patients due to the age threshold of the study group and the COVID-19 pandemic. Therefore, larger further studies are required to validate our findings. Another limitation is the upper limit of age used (51 years), the typical definition of young STEMI being under 64 years (young and middle aged) or under 45 (young). We used a non-invasive method to determine VAC (even though it is a validated method, the invasive measurement remains the gold standard). Another limitation would be the short follow-up period (one year) and the fact that we only had a telephonic follow-up available due to COVID-19 pandemic restrictions. 

Despite these limitations, our study holds great potential, considering the obtained results and their further possible utility in current clinical practice, and with potential utility in postinfarction risk stratification in young patients.

## 6. Conclusions

The 3D ventricular-arterial coupling proved to be an independent predictor for MACE at 12 months after STEMI in young patients having incremental prognostic value over LVEF and LVGLS. Larger scale studies are needed to validate these findings.

## Figures and Tables

**Table 1:** General characteristics of the study group.

	Study Population (*n* = 84)	MACE Group (*n* = 13)	Non-MACE Group (*n* = 71)	*p* Value
Clinical characteristics				
Age (years)	44 ± 4.62	46.54 ± 4.59	44.35 ± 5.67	0.244
Killip class ≥ 2	10.7%	50%	1.4%	**<0.0001**
Cardiovascular risk factors				
Smoking	86.9%	77.8%	87%%	0.370
Obesity	13.1%	9.1%	14.1%	0.546
Hypertension	45.2%	45.5%	44.4%	0.600
Dyslipidaemia	83.3%	90.9%	81.9%	0.446
Diabetes	14.1%	25%	11.4%	0.199
Positive family history	36.9%	41.7%	36.6%	0.756
Angiographic characteristics				
LAD	48.8%	75%	43.7%	0.08
RCA	32.1%	25%	33.8%	
LCX	19%	0%	22.5%	
Multivessel disease	34.5%	27.3%	34.7%	0.545
Occluded artery	583%	54.5%	58.3%	0.531
PCI over 12 h	15%	40%	60%	0.216
TIMI post PCI				
0–2	7.1%	0%	8.5%	0.504
3	92.9%	83.3%	91.5%	
Laboratory characteristics				
WBC count, ×10^3^/mm^3^	13,541.9 ± 11,965.64	15,790.91 ± 3437.57	13,140.56 ± 12,826.55	0.499
Haemoglobin, g/dL	14.5 ± 2.04	13.53 ± 1.05	14.66 ± 2.13	0.087
Creatinine (mg/dL)	0.84 ± 0.208	0.91 ± 0.32	0.82 ± 0.17	0.372
Glycemia (mg/dL)	116.47 ± 41.09	154.42 ± 67.91	109.63 ± 31.07	0.05
Cholesterol (mg/dL)	232.19 ± 59.61	222.33 ± 71.14	234.47 ± 58.04	0.519
Triglycerides (mg/dL)	183.62 ± 142.55	208.08 ± 245.91	180.18 ± 119.40	0.228
HDL-cholesterol	160.73 ± 52.21	147.84 ± 63.52	163.6 ± 50.5	0.421
LDL-cholesterol	26.97 ± 12.08	26.47 ± 12.3	27.56 ± 11.9	0.806
Peak CK-MB (U/L)	238.45 ± 192.250	427.76 ± 278.66	206.76 ± 156.35	**0.020**
GRACE score	86.93 ± 15.29	104 ± 23.66	84.04 ± 11.3	**0.014**
Medication at discharge				
Dual antiplatelet therapy	100%	100%	100%	0.713
Beta-blockers	95.2%	92.3%	95.7%	0.496
ACEI/ARB	96.42%	92.3%	97.2%	0.578
Statins	100%	100%	100%	0.713

LAD—left anterior descending artery, RCA—right coronary artery, LCX—left circumflex artery, WBC—white blood cells; ACEI—angiotensin-converting enzyme inhibitors; ARB—angiotensin receptor blockers; Bolded *p* values are statistically significant.

**Table 2:** Echocardiographic parameters.

	Control Group *n* = 28	STEMI *n* = 84	MACE *n* = 13	Non-MACE *n* = 71	*p*-Value MACE/ Non-MACE
Systolic function					
2D LVEDV (mL/mp)	48.23 ± 9.4	53.01 ± 13.24	63.96 ± 14.48	51.16 ± 12.18	**0.002**
2D LVESV (mL/mp)	21.17 ± 5.32	30.71 ± 10.64	43.98 ± 14.48	28.47 ± 8.47	**0.001**
2D LVEF (%)	56.35 ± 4.8	42.19 ± 7.34	35.75 ± 5.81	43.61 ± 6.608	**<0.0001**
3D LVEDV (mL/mp)	52.3 ± 11.3	57.1 ± 13.89	68.29 ± 13.14	55.22 ± 13.14	**0.002**
3D LVESV (mL/mp)	22.9 ± 7.2	32.91 ± 11.01	46.28 ± 12.49	30.65 ± 9.03	**0.001**
3D LVEF (%)	40.14 ± 14.71	43.17 ± 7.74	37.16 ± 6.307	44.88 ± 6.605	**<0.0001**
3D SV (mL)	56.07 ± 5.54	46.54 ± 10.16	40.22 ± 7.57	48.55 ± 9.55	**0.013**
3D SV (mL/mp)	40.02 ± 7.8	24.13 ± 6.27	20.1 ± 7.5	27.6 ± 9.4	**0.017**
LVGLS	−20.98 ± 4.5	−13.21 ± 3.47	−8.9 ± 2.51	−13.95 ± 3.066	**<0.0001**
LV mechanical dispersion (ms)	30.8 ± 7.9	68.57 ± 25.81	88.28 ± 27.67	65.25 ± 24.13	**0.004**
Diastolic function					
E (m/s)	0.83 ± 0.15	0.71 ± 0.21	0.68 ± 0.202	0.72 ± 0.21	0.564
A (m/s)	0.64 ± 0.14	0.65 ± 0.14	0.62 ± 0.14	0.66 ± 0.13	0.4
E/A	1.35 ± 0.37	1.14 ± 0.45	1.16 ± 0.52	1.14 ± 0.44	0.863
E/e’ (LV filling pressures)	6.19 ± 1.62	8.03 ± 2.29	10.68 ± 7.59	7.59 ± 2.03	**<0.0001**
2D LA volume (mL)	35.67 ± 11.98	45.37 ± 13.25	54.16 ± 18.21	43.88 ± 11.57	0.052
2D LA volume (mL/m^2^)	19.76 ± 9.87	23.85 ± 7.29	29.82 ± 10.77	22.85 ± 6.08	**0.027**
Diastolic dysfunction grade					
0	100%	29.3%	0%	34.3%	**0.009**
1	0%	58.5%	66.7%	57.1%	
2	0%	12.2%	33.3%	8.6%	
3	0%	0%	%	0%	
RV function					
RV-RA gradient	16.1 ± 6.73	23.52 ± 13.42	24.45 ± 14.1	22.53 ± 13.41	0.88
TAPSE (mm)	23.6 ± 6.8	18.55 ± 5.93	16.58 ± 6.08	18.88 ± 5.88	0.215
S’ (m/s)	0.15 ± 0.02	0.12 ± 0.024	0.12 ± 0.02	0.1 ± 0.023	0.133

Bolded *p* values are statistically significant.

**Table 3:** Univariate COX regression for MACE prediction.

Univariate Analysis	Parameters	HR	95% CI	*p*
Clinical characteristics	Age	1.060	0.954–1.178	0.272
	Sex	0.602	0.132–2.750	0.536
	Killip class over II	0.04	0.012–0.130	**<0.0001**
Echocardiography parameters	**2D LVEF**	0.826	0.751–0.910	**<0.0001**
	**2D LVEDV indexed**	1.085	1.042–1.150	**<0.0001**
	**2D LVESV indexed**	1.140	1.083–1.2001	**<0.0001**
	**LVGLS**	1.778	1.359–2.325	**<0.0001**
	**3D LVEF**	0.787	0.708–0.874	**<0.0001**
	**3D LVEDV indexed**	1.090	1.037–1.146	**<0.0001**
	**3D LVESV indexed**	1.126	1.070–1.184	**<0.0001**
	**E/e’**	1.659	1.289–2.134	**<0.0001**
	**2D LAVi**	1.120	1.050–1.194	**0.002**
	3D EA	1.972	0.805–4.826	0.145
	**3D EES**	0.025	0.004–0.172	**<0.0001**
	**3D VAC**	13.827	5.247–36.437	**<0.0001**
Laboratory parameters	**Peak CK-MB**	1.004	1.002–1.006	**<0.0001**
	Leukocytes	1	1.000–1.000	0.534
	Haemoglobin	0.698	0.487–1	0.06
	Glycemia	1.015	1.006–1.023	0.09
	Creatinine	6.658	0.414–107.164	0.181
	Cholesterol	0.996	0.986–1.006	0.394
	Trigliceryde	1.001	0.997–1.005	0.569

HR—hazard ratio; CI—confidence interval; Bolded variables and *p* values are statistically significant.

**Table 4:** Multivariable (Adjusted) Cox regression analysis for MACE.

3D VAC + Regression Models	HR (95% CI)	*p*
3D VAC	13.82 (5.2–36.43)	**<0.0001**
+Model 1 (Killip class)	5.69 (1.46–22.1)	**0.012**
+Model 2 (E/e’)	8.68 (2.88–26.17)	**<0.0001**
+Model 3 (peak CK-MB)	9.12 (3.11–26.78)	**<0.0001**
+Model 4 (Killip class, E/e’, CK-MB)	9.66 (0.7–28.32)	**0.002**
+Model 5 (LVEF)	12.004 (2.21–65.12)	**0.004**
+Model 6 (LVGLS)	5.23 (1.5–17.94)	**0.009**
+Model 7 (Killip class, E/e’, CK-MB, LVEF)	8.403 (3.2–45.3)	**0.004**
+Model 8 (Killip class, E/e‘, CK-MB, LVGLS)	7.37 (0.42–8.26)	**0.007**

Bolded *p* values are statistically significant.

**Table 5:** VAC parameters by 2D and 3D echocardiography.

Variable	2D Echocardiography	3D Echocardiography	*p* Value
EA	2.4 ± 0.58	2.38 ± 0.59	0.077
EES	1.92 ± 0.67	1.85 ± 0.57	0.06
VAC	1.32 ± 0.52	1.36 ± 0.48	0.136

## Data Availability

Data are available upon reasonable request.

## Supplementary Materials

The following supporting information can be downloaded at: https://www.mdpi.com/article/10.3390/biomedicines12010105/s1, Table S1: Correlations between VAC and other echocardiographic parameters; Table S2: Ventricular-arterial coupling parameters.

## Abbreviations

ACEI	Angiotensin-converting enzyme inhibitors
AMI	acute myocardial infarction
ARB	Angiotensin receptor blockers
AUC	area under the curve
BP	blood pressure
CABG	Coronary artery bypass grafting
CAD	coronary artery disease
CK-MB	creatine kinase-myocardial band
CI	confidence intervals
CO	cardiac output
DCM	dilated cardiomyopathy
EA	arterial elastance
EES	left ventricular end-systolic elastance
EF	ejection fraction
ENd (avg)	averaged normalized ventricular elastance at the onset of ejection
ENd (est)	estimated normalized ventricular elastance at the onset of ejection
ESP	end-systolic pressure
HCM	hypertrophic cardiomyopathy
HDL	high-density lipoprotein
HF	heart failure
LA	left atrium
LAD	left anterior descending artery
LCX	left circumflex artery
LDL	low-density lipoprotein
LAVi	left atrial indexed volume
LV	left ventricle
LVEDV	Left ventricular end-diastolic volume
LVEF	left ventricular ejection fraction
LVESV	Left Ventricular End-Systolic Volume
LVGLS	left ventricular global longitudinal strain
MACE	major adverse cardiac events
NTproBNP	N-terminal pro B-type natriuretic peptide
NYHA	New York Heart Association
PCI	percutaneous coronary intervention
PWV	pulse-wave velocity
RA	right atrium
RAAS	Renin-Angiotensin-Aldosterone System
RCA	left coronary artery
ROC	receiver operating characteristic
RV	right ventricle
STEMI	ST elevation myocardial infarction
SV TAPSE	stroke volume Tricuspid Annular Plane Systolic Excursion
tND	ratio of the pre-ejection period to the total systolic period measured on the aortic pulse
VAC	ventricular arterial coupling
WHC	white blood cells
WMSI	Wall motion score index
