# Supplementary material for: Left Ventricular-Arterial Coupling as an Independent Predictor of Adverse Events in Young Patients with ST Elevation Myocardial Infarction—A 3D Echocardiographic Study
Source: Biomedicines. 2024 Jan 4;12(1):105. doi: 10.3390/biomedicines12010105 (PMC10812951; doi:10.3390/biomedicines12010105)
Supplement: Supplementary file 1 [file biomedicines-12-00105-s001.zip › biomedicines-2792402-supplementary.pdf]

SUPPLEMENTARY MATERIALS

Table S1. Correlations between VAC and other echocardiographic parameters.

**Correlations**				
		EA	EES	VAC
**2D LVEF**	Pearson Correlation	-0.423**	0.523**	-0.862**
	P value	**<0.0001**	**<0.0001**	**<0.0001**
**2D LVEDV**	Pearson Correlation	-0.283*	-0.626**	0.443**
	P value	**0.01**	**<0.0001**	**<0.0001**
**2D LVESV**	Pearson Correlation	0.002	-0.693**	0.750**
	P value	0.985	**<0.0001**	**<0.0001**
**3D LVEF**	Pearson Correlation	-0.458**	0.559**	-0.918**
	P value	**<0.0001**	**<0.0001**	**<0.0001**
**3D LVEDV**	Pearson Correlation	-0.342	-0.714**	0.475**
	P value	**0.002**	**<0.0001**	**<0.0001**
**3D LVESV**	Pearson Correlation	-0.004	-0.734**	0.781**
	P value	0.970	**<0.0001**	**<0.0001**
**LVGLS**	Pearson Correlation	.471**	-0.381**	0.772**
	P value	**<0.0001**	**<0.0001**	**<0.0001**
**LV dispersion**	Pearson Correlation	.289**	-0.068	0.347**
	P value	**0.008**	0.539	**0.001**

** Correlation is significant at the 0.01 level. * Correlation is significant at the 0.05 level. Bolded p values are statistically significant.

Table S2. Ventricular-arterial coupling parameters.

	**Control group N=28**	**STEMI N=84**	**P**	**MACE N=71**	**Non MACE N=13**	**P**
EA	2.1±0.6	2.38±0.59	**<0.0001**	2.33±0.59	2.65±0.54	0.088
EES	2.3±0.7	1.85±0.57	**<0.0001**	1.95±0.55	1.208±0.52	**<0.0001**
VAC	0.8±0.23	1.37±0.48	**<0.0001**	1.27±0.37	2.2±0.59	**<0.0001**

Abbreviations are in text. Bolded p values are statistically significant.
